# Depression Related to COVID-19, Coping, and Hopelessness in Sports Students

**DOI:** 10.3390/brainsci14060563

**Published:** 2024-05-31

**Authors:** Laura Rodica Giurgiu, Cosmin Damian, Anca Maria Sabău, Tudor Caciora, Floricica Mariana Călin

**Affiliations:** 1 Department of Psychology and Education Sciences, Faculty of Psychology and Education Sciences, Ovidius University of Constanta, 900527 Constanta, Romania; laura.giurgiu@365.univ-ovidius.ro (L.R.G.); mariana.calin@365.univ-ovidius.ro (F.M.C.); 2 Department of Physical Education, Sport and Kinetotherapy, Faculty of Physical Education and Sport, Ovidius University of Constanta, 900527 Constanta, Romania; cosmin.damian@ymail.com; 3 Department of Physical Education, Sport and Kinetotherapy, Faculty of Geography, Tourism and Sport, University of Oradea, 410087 Oradea, Romania; sabauancamaria@yahoo.com; 4 Department of Geography, Tourism and Territorial Planning, Faculty of Geography, Tourism and Sport, University of Oradea, 1 Universitatii Street, 410087 Oradea, Romania

**Keywords:** COVID-19, sport students, depression, coping mechanisms

## Abstract

This study aimed to explore the impact of the first two waves of the COVID-19 pandemic on the mental and physical states of sports students from Romania and also to compare the differences according to gender and the type of sport. Initially, in order to collect demographic data and health reports, a cross-sectional survey was developed to evaluate the emotional needs of sports students during the pandemic. After the second wave, the coping strategies used by the participants to fight negative emotions were assessed using the CERQ questionnaire. The results indicate that depression symptoms are the most reported psychological issues among the participants and that there are differences according to gender concerning the cognitive schemas they use in order to reduce the symptoms. Also, it was found that there are differences, corresponding to the type of sport, in choosing adaptive coping mechanisms. Ultimately, it was confirmed that higher levels of hopelessness among sports students are associated with increased vulnerability to substance use, with the correlation between those two indicators being strong. Delving deeper into this relationship can help identify critical points for intervention to prevent substance abuse. At the same time, the dichotomic analysis of the results found as moderators—the gender and the type of sport in decreasing the severity of depression could be an important aspect of the next counseling interventions.

## 1. Introduction

Even before facing COVID-19, infection experts warned of the rising threat of new, highly dangerous viruses, as well as the human body’s increased resistance to antibiotics and lowered immunity. Any pandemic is an existential crisis that threatens individuals’ health and lives. People who have experienced disease can react in a variety of ways, ranging from indifference to fatalism, from denying risks and failing to follow sanitary norms to intense anxiety, panic, or even progressive cognitive and emotional decline [1,2,3]. Substance abuse and binge eating are examples of unhealthy stress coping mechanisms [4,5]. On the other hand, adaptive strategies may include a personal routine, communicating with others online, and focusing on any potential benefits of the situation, which changes one’s perspective on life’s priorities [6]. As clinical manifestations in extreme and torturous environments, exhaustion, social withdrawal, guilt-related sleep problems, helplessness and despair, emotional confusion, and suicidal thoughts can occur. These varied reactions to the pandemic, such as increased stress, different coping strategies, and psychological impacts, are directly related to the research objectives and assumptions by emphasizing the importance of investigating these specific responses among sports students, thus providing insights into their unique challenges and informing targeted mental health interventions. When the trauma cannot be integrated into the person’s cognitive schemes, the individual may enter the discharge phase or a traumatic process. The scheme has been referred to, in the clinical context, as a framework that the person uses to scan, encode, and assess stimuli that have the potential to mobilize or demobilize the body since Beck in 1967 [7].

A widely accepted definition of coping is a person’s efforts to deal with requirements that are assessed as exceeding or overloading his resources [8,9,10]. Coping skills are components of the subjective response to stressful life events, and cognitive factors such as attributions, expectations, beliefs, and attitudes play a significant role in determining whether or not a person will act when they are feeling uncertain or defeated [11,12]. The unconscious cognitive processes, such as projection or denial, are distinguished from the conscious cognitive processes, which include self-blame, blaming others, rumination, and catastrophic thinking. For most authors, the problem-centered coping strategies are thought to be more useful than the emotion-centered ones [13,14,15]. 

It is now widely acknowledged that COVID-19 is a threat to life, health, and sports careers. The pandemic has resulted in significant financial losses for the sports industry as a consequence of the cancellation of major international competitions, including the 2020 Tokyo Olympic Games. It is well known that athletes suffer not only financial losses but also significant physical, nutritional, and psychological harm, affecting their competitive and performance capacity [16,17]. Thus, Makarowski et al. (2020) [18] state that the pandemic had no serious effect on the level of psychological stress among athletes because behavioral disengagement allowed the athletes’ subjective stress to be reduced. This is because the great majority of them used adaptive coping strategies that lowered their subjectively experienced stress, and the close cooperation of athletes with a coach and/or a sports psychologist minimized daily stressors for them.

The postponement of the Olympic Games represented a significant interruption in the career of many Olympic athletes, resulting in a loss of identity, motivation, and purpose, as well as emotional responses that can activate mental health disorders [19,20]. Elite athletes reported sleep disorders, depression, anxiety, post-traumatic stress, eating problems, attention deficit or hyperactivity, substance use, pathological chance gaming, or other addictions [21]. It appears that many elite athletes avoid discussing these topics with friends and family because they feel pressured to appear strong and show no signs of weakness. In times of normality, the causes of depressive symptoms could be the loss of devotion and commitment to competition following an injury or retirement from sports [22,23]. Research conducted during the quarantine and restriction effects showed that the current generation of Olympic athletes has suffered from a lack of perspective, and only a few of them have received professional help [24,25].

It is interesting to point out that once the athletes returned to stadiums, another challenge appeared: the so-called ‘ghostly game’ (to play matches without the attendance of supporters, with empty tribunes). Certain researchers [26] took advantage of this once-in-a-lifetime opportunity to study the effects of public absence on the behavior of professional football players. The behavior of the Austrian players was analyzed and compared in 20 “ghosts’ games.” Marlovits [27] discovered that the most difficult challenge for players was “adapting to a non-resonant environment”. Athletes all over the world struggled to make the transition from isolation to practice and competition, and they were eager to compete, even without a public, but televised [28,29].

The negative effects of isolation resulted in a lot of athletes experiencing insomnia and depression, decreased body mass and increased body fat percentage, loss of mental clarity and tenacity, and a lack of relationships with families and loved ones that exacerbated the stress manifestation [30,31]. In these circumstances, athletes reacted differently according to gender. Women reported higher levels of perceived stress and dysfunctional psycho-biosocial states than men, but according to Garnefski [32], women use it more often. Positive refocusing means thinking about joyful and pleasant issues rather than the actual negative event. Both male and female students reported using substances in order to cope, but the female athletes, by maintaining social networks and being willing to talk about their emotions, may have discovered coping strategies to offset the extra pressures of the pandemic [33,34,35].

According to the type of sport practiced, athletes reported different mental health issues during the lockdown. Team athletes reported more insomnia and depression symptoms than individual sport athletes, possibly as a result of new and unfamiliar social isolation and a lack of social team activity [36,37]. Individual athletes are more likely to develop psychological problems in typical conditions because performing well in individual sports requires a higher level of internal attribution and no opportunity to share responsibility with teammates [38]. 

During the lockdown, individual athletes reported fewer psychological changes than team athletes because they usually had to train alone in the absence of coaches and colleagues [39,40]. The prevalence of sadness and depression in team sports may be higher due to the absence of social interactions with teammates and technical staff, which has resulted in anxiety and uncertainty about many aspects of their lives [41,42,43]. 

Optimism has played an important role in dealing with quarantine-related detention, but finally, the lack of hope associated with limitations and a decline in the quality of people’s lives has resulted in an increase in anxiety, panic, and insomnia [44,45,46]. The study of strategies used by athletes to overcome adversity during COVID-19 led to identifying the consumption of psychoactive substances, which appears to have a significant impact on their mood but, in the long run, increases the need for consumption [47]. 

The isolation aggravated by the loss of autonomy and changes in the sports environment and in personal life, including early retirement from the sport, results in the use of alcohol, smoking, and drugs with sedative effects at the limit of violation of anti-doping policies [48,49]. Even so, the findings showed that substance abuse was generally at a lower level in athletes than in non-athletes [50].

Taking into account the above, in our paper, we aimed to show, on the one hand, the emotional manifestations of sports students during the pandemic’s first two waves and to emphasize the coping strategies they used to fight stressful situations, compared to gender and the type of sport. As specific objectives of this study, we are aiming to highlight the emotional issues that the participants experienced during COVID isolation. At the same time, by performing a comparative analysis based on gender regarding the psychological states and moderating by the cognitive–emotional response, we consider testing the hypotheses according to which there are gender differences regarding the coping mechanisms intended to reduce the depression symptoms; the second hypotheses presume that there are differences depending on the type of sport, in terms of cognitive schemas, for reducing depression.

These studies can also offer deep insights into the long-term psychological effects and provide valuable lessons for handling future crises. By understanding the varied impacts based on gender and sport type, we can enhance mental health support systems tailored specifically to athletes’ needs. This not only has the potential to help in developing robust interventions for future public health emergencies but can also contribute to the academic field by guiding policy development and improving training programs. Additionally, such research can support the normalization and destigmatization of mental health issues within the sports community, encouraging open discussions and proactive seeking of support. This nuanced approach not only enriches the current understanding of mental health in athletic populations during crises but also contributes significantly to the existing literature by highlighting the importance of targeted mental health interventions. The findings offer valuable implications for developing tailored support systems that can enhance psychological resilience and well-being among sports students in future public health emergencies.

## 2. Materials and Methods

The research was conducted over the first two waves of COVID-19 in Romania between March and November 2020. Following our online classes during the lockdown, as well as mentoring students at risk, we collected primary reports on the physical and mental states they encountered during the restriction period. Considering these reports and the frequency and intensity of the feelings and moods, we developed and validated our survey regarding the emotional needs of sports students during the lockdown. 

After resuming the competition season with no spectators in the stands, the decision was taken to explore deeper into the athletes’ coping strategies in order to understand what helped them reduce emotional and cognitive stress. Thus, during the second pandemic wave in Romania (November 2020), we applied the CERQ (Cognitive Emotional Coping Questionnaire). Coping questionnaires are psychological tools used to understand how individuals respond to stress and challenges. Coping refers to conscious efforts to manage psychological demands or stress. These strategies can range from adaptive approaches that help the individual cope effectively to less adaptive approaches that can exacerbate problems or stress [51,52,53]. The application of such a questionnaire in this case, where our study focuses on athletes, is all the more relevant. Considering that athletes often face high levels of psychological and physical stress, the ways in which they manage these pressures can significantly influence their performance, and the COVID-19 pandemic and the restrictions imposed by it act as an additional stress factor for most athletes [18].

The target group chosen for the analysis was decided to be made up of students who are also athletes. This choice is due to several reasons. Applying a coping questionnaire to student-athletes during a COVID-19 lockdown is highly beneficial. It helps understand the unique stress caused by the disruption of both academic and athletic routines. Insights from the questionnaire can guide the creation of tailored support systems, addressing the dual challenges these individuals face. Additionally, understanding their coping mechanisms can assist in maintaining their performance and mental health during crisis situations. This approach not only aids in immediate support but also helps in building resilience for future challenges, ensuring holistic support for their academic and athletic needs [54,55].

Initially, 146 sports students from across Romania, from various sports disciplines distinguished by remarkable athletic performances, were recruited for the target group. Out of the 146 athletes initially targeted, only 103 responded positively and completed the questionnaire. The target group was chosen to ensure a balance between genders (male and female), types of sports (individual and team), levels of achievement (national and/or European champions), and levels of proficiency (medium or lower levels). This approach was designed to include a structurally diverse and varied sample of athletes, aiming to accurately determine the implications of COVID-19 for them. No partial responses were recorded, and no defining characteristics were identified for the group that did not respond to the questionnaire. The selection methodology aimed to mitigate any potential biases that might arise from a less balanced sample. By ensuring a diverse group of participants, this study seeks to provide a comprehensive overview of how COVID-19 has impacted athletes from different backgrounds and levels of achievement. Considering the impossibility of meeting the respondents in person, a Google Form link was open for two weeks in May 2020 for the survey and two weeks in November 2020 for CERQ, and the duration of the completion was about 20 min for each participant. 

In our study, the subgroups for comparative analysis were structured based on several key criteria to ensure a detailed exploration of how various factors influence coping mechanisms. These criteria included age, gender, type of sport, duration of practice, personal best results, and competitive classification. The level of competition among these athletes varied from national to international levels, adding a layer of complexity to the coping strategies that might be employed under different pressures. More than 60 of the subjects were multiple national champions, indicating a high level of proficiency and experience in their respective sports. Furthermore, twenty of these athletes had extensive international experience, having participated in European tournaments and world championships. This distinction is crucial as it highlights their exposure to high-stakes environments, which could influence their psychological resilience and coping styles. This detailed structuring of subgroups allows for nuanced comparative analyses, giving insights into how different variables may interact to influence the coping mechanisms of student-athletes facing the dual challenges of academic and athletic pressures, especially during the heightened stress of a global pandemic.

In order to collect the demographic data and health reports, a cross-sectional survey was developed regarding the emotional needs of sports students during the pandemic. Following discussions with student-athletes during online group and individual meetings, emotion and state logs were created to document the feelings and experiences frequently reported by the students. There were formulated 22 items, organized in five subscales, regarding psychological states like fear, sadness, nervousness, or hopelessness; general health indicators, such as sleep quality, food disorders, physical condition, substances` use, or dietary supplements; type of relationships with family, friends, or colleagues; social media and substance use; perceived risks and uncertainty in the academic and professional field, etc. The 22 items’ responses were distributed on a Likert scale of 5 points, ranging from 1 (not at all) to 5 (always). Applying a comprehensive questionnaire with 22 items across five subscales offers a multidimensional approach to understanding the impact of a crisis on individuals. By assessing psychological states like fear and hopelessness as well as general health indicators, including sleep quality and substance use, this tool helps identify key areas of mental and physical health that may require intervention. Additionally, insights into social dynamics and the use of social media provide a deeper understanding of respondents’ social support systems and coping mechanisms. Evaluating perceived risks in academic and professional environments also helps organizations tailor their support and communication strategies effectively. 

To ensure the robustness and reliability of the questionnaire, a test–retest validation method was employed. This method involves administering the same questionnaire to the same group of participants at two different points in time. By comparing the results from the two administrations, researchers can assess the consistency and stability of the responses over time. A high correlation between the two sets of responses indicates that the questionnaire reliably measures what it is intended to measure. To ensure the reliability and validity of the study, rigorous screening processes were implemented. Participants were assessed for pre-existing mental health conditions through comprehensive self-reported medical histories and psychological assessments, excluding those with severe pre-existing disorders. Socioeconomic conditions were thoroughly evaluated, collecting detailed data on education and living conditions, which were included in the statistical models to control for their influence on mental health outcomes. Age was systematically controlled by stratifying participants into distinct age groups, with age-adjusted analyses performed. 

In addition to test–retest validation, the items were also reviewed by experts in psychology and sports science to ensure they accurately captured the relevant emotional and psychological states. This expert review process provided valuable feedback on the clarity and relevance of each item, leading to further refinements. Pilot testing with a small sample of student-athletes was also conducted to identify any potential issues with the wording or interpretation of the items. Based on the feedback from these stages, adjustments were made to enhance the accuracy and comprehensibility of the questionnaire. The final set of 22 items thus represents a carefully validated tool capable of providing detailed and reliable insights into the emotional and psychological states of student-athletes during the COVID-19 pandemic. This tool is essential for understanding the impact of the pandemic on their well-being and for developing targeted interventions to support them. Overall, this detailed questionnaire facilitates data-driven decision-making in crisis situations, enabling stakeholders to implement targeted and effective support measures that address both the immediate and long-term needs of their communities during challenging times.

The Cognitive Emotion Regulation Questionnaire (CERQ) was applied to identify the coping strategies used by participants to manage negative emotions. This tool, comprising 36 items measuring nine distinct strategies, such as self-blame, acceptance, and positive reappraisal, provides crucial insights into how individuals cope with stress and adversity. By determining the effectiveness of these strategies, the questionnaire helps in assessing their impact on mental health, enabling tailored psychological interventions. It facilitates a comparative analysis across different groups, enhancing our understanding of emotional regulation. Ultimately, this detailed insight helps professionals design better support mechanisms and aids participants in improving their own coping techniques, promoting better mental health outcomes [56,57].

After obtaining and integrating the results, the statistical data analysis was performed using IBM SPSS Statistics 26 and Matlab 7. The responses collected from both instruments were analyzed dichotomously, aiming to find the moderator variables of the adaptive behavior manifested by the participants. In this study, we implemented a comprehensive array of statistical analyses to rigorously evaluate the data collected through the Cognitive Emotion Regulation Questionnaire. The use of descriptive statistics such as the arithmetic mean, median, standard deviation, and coefficient of variability helped in summarizing the central tendencies and variability of the coping strategies used by participants. This provides a clear picture of typical responses and the spread of data, which is essential for understanding general coping behavior patterns.

Factorial analysis was employed to uncover THE underlying dimensions of coping, identifying how various strategies cluster together, which is crucial for developing targeted psychological interventions. ANOVA was used to compare differences in coping strategies across different demographic groups, helping to pinpoint specific subgroups that might benefit from tailored support. An ANOVA was employed to compare the mean differences between different groups (e.g., gender, type of sport) in terms of various psychological outcomes. This test is appropriate for our study because it helps determine whether there are statistically significant differences in mental health outcomes across different groups of sports students, thus providing insights into how different factors influence these outcomes.

Spearman correlation and regression analyses were conducted to explore the relationships between different coping strategies and psychological outcomes, like stress or mental health. These analyses allow us to assess the strength and direction of these associations, providing valuable insights into how different coping mechanisms affect mental health and thus informing better practices for mental health support. Together, these statistical tools enable a deep, nuanced understanding of coping strategies, guide effective interventions, and contribute significantly to the field of psychological resilience research.

These statistical tests were selected based on their suitability for the type of data collected and the research questions posed. By employing a combination of multivariate regression analysis, ANOVA, and Spearman correlation, we can gain a comprehensive understanding of the relationships and differences within our data, thereby ensuring robust and reliable findings.

This study conducted five types of analyses based on the collected survey data: characteristics of the target group, factorial analysis, correlation analysis, variations in coping mechanisms by gender, and variations in depression and cognitive schemas by sport type. These analytical approaches complement each other effectively, providing a precise understanding of cause–effect relationships. They also detail the levels of stress and depression induced by the COVID-19 pandemic, varying according to the characteristics of the respondents.

## 3. Results

### 3.1. Target Group Characteristics

**Figure 1:**
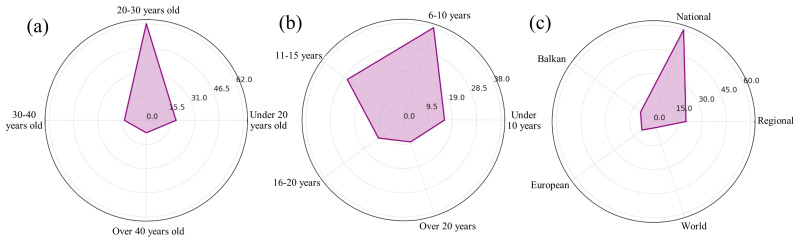
The characteristics of the target group chosen for completing the questionnaire ((**a**)—distribution of athletes according to age; (**b**)—sports experience of the subjects; (**c**)—level of competitions won by the subjects).

The participants consisted of 103 student-athletes, split nearly evenly by gender, with 52 women and 51 men participating. The age range of the participants was from 19 to over 40 years, which provided a broad perspective across different maturity levels in both sports and academics. The majority of athletes (approximately 60%) are in the age group of 20–30 years, closely followed by the segment under 20 years of age (approximately 18%), which shows that a significant segment is composed of young athletes. The 30- to 40-year-old category represents approximately 14% of the total, suggesting that athletes remain active in competitions at relatively advanced ages. Only 8% of athletes are over 40 years old (Figure 1a). These student-athletes were involved in a wide array of sports disciplines, categorized into individual sports and team sports. Specifically, 44 were from individual sports such as athletics and swimming, while 59 were team players participating in sports like football and basketball.

Experience in sports is a key indicator of the performance achieved by athletes. The majority of athletes (37%) fall into the 6–10 year experience category, a sign that many of them reach high levels of performance after years of practice and dedication. Another important segment (26%) includes athletes with 11–15 years of experience, reflecting a long-term commitment essential to excellence in sport. Also, 16% of the athletes have less than 5 years of experience, which suggests the presence of emerging talents who are rapidly progressing in their careers. Athletes with experience between 16 and 20 years and those with more than 20 years of experience represent 12% and 9%, respectively, indicating a tendency to withdraw from top competitions with the accumulation of years and experience (Figure 1b). This distribution underscores the fact that longevity in sports is often balanced by new influxes of talent. 

Regarding the competitions in which they obtained notable results, approximately 58% of the athletes triumphed in national competitions, demonstrating a high level of success and competence on the domestic stage. Regional and Balkan champions are less common, with 19% and 10% of the total, respectively, indicating that although a significant number of athletes achieve victories at the national level, fewer manage to advance to larger competitions. At the same time, participation and success in European and world competitions are even rarer, with only about 9% and 4%, respectively (Figure 1c). It highlights the exceptional performances of a select group of athletes who distinguish themselves on the international stage.

### 3.2. Factorial Analysis 

**Figure 2:**
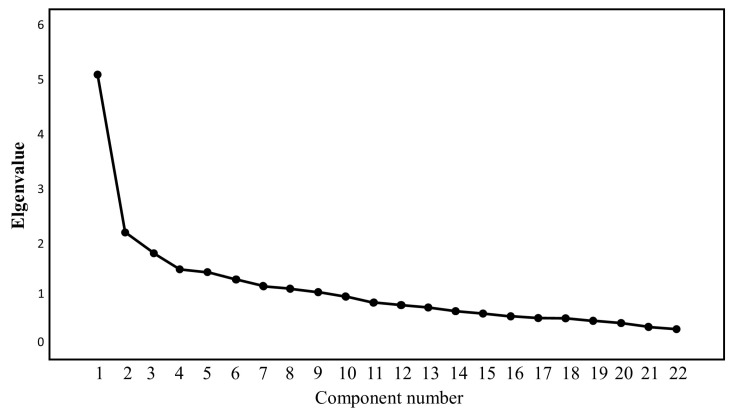
Graphical representation of score distribution of the items by varimax rotation.

In order to find the factorial structure of the survey, varimax rotation was the method of choice. Table 1 shows that the model obtained from the 22 items explains 46.7% of the variance. This indicates that the factors identified through the analysis explain nearly half of the variability observed in the participants’ responses. In other words, 46.7% of the differences in the responses can be attributed to the factors included in the model, suggesting a significant but not complete explanation of the data’s variability. As seen from Figure 2, factor 1 is the most important by visual inspection to be taken into consideration, followed by the other five factors (with their own value bigger than 1), which justifies keeping them in the model. 

It can be seen in Table 1 how the items of the survey are organized based on the three selected factors. Thus, for Factor No. 1—“depression”, the most significant items are 1, 2, 3, 4, 5, and 12. Factor No. 2—“loneliness and fear”—is composed of items 16, 19, 20, and 21. For Factor No. 3—“substance consumption”, it is noticed that items 13, 14, and 22 are negatively charged. The three selected factors were identified using factor analysis, specifically Principal Component Analysis with varimax rotation, to group related variables based on their correlations. This statistical method reduces the number of variables by identifying underlying factors that explain the patterns in the data.

The analysis of the relevant survey responses clearly highlights the profound impact that the COVID-19 pandemic has had on the mental state of individuals. Factor 1, which focuses on pandemic-related depressive symptoms, includes items that directly reflect heightened feelings of anxiety and depression during the global health crisis.

From factor 1, item 4 (“Since the start of the pandemic, I have been nervous”) has the highest loading on this factor (0.791), signaling a direct and strong link between pandemic events and increased levels of anxiety. This shows that the uncertainty and rapid changes in everyday life had an immediate and visible impact on emotional well-being. Item 3 (“I’ve been feeling depressed lately”) and Item 1 (“Since the pandemic, I feel hopeless”) have loadings of 0.758 and 0.663, respectively. These responses emphasize acute feelings of hopelessness and a deep depressive state, reflecting how extensive conditions of isolation and constant health and economic anxiety can degrade mental health. Articles 5 and 12 address the indirect impacts of the pandemic. Item 5 (“Watching the news about COVID makes me anxious”), with a loading of 0.573, illustrates how continued exposure to news about the pandemic can exacerbate feelings of anxiety and agitation. On the other hand, item 12 (“I had difficulty sleeping during this period”), with a loading of 0.412, indicates sleep-related problems, a common symptom among those experiencing increased stress and anxiety. These not only reflect direct responses to immediate stress but also how prolonged stress can disrupt vital functions such as sleep, contributing to an overall decline in quality of life (Table 1).

Cronbach’s alpha index of 0.763 showed the internal consistency of depression related to the pandemic. Cronbach’s alpha index shows satisfactory fidelity for Factor 1 regarding the internal consistency of our survey. A pattern of coping mechanisms has been shown to be predictable for depression related to the pandemic caused by the COVID-19 pandemic, but the relationship is moderated by gender and sport type (individual versus team sport).

Factor 2 of the survey’s factor analysis, which focuses on loneliness and fear, brings to the fore items that highlight feelings of anxiety and isolation experienced by respondents in the context of the COVID-19 pandemic. This factor comprises several significant articles that together illustrate the complexity of the pandemic’s emotional impact on various aspects of daily life. Item 21, “I am concerned about being tested for COVID-19”, with a loading of 0.789, reflects a deep fear about the possibility of being infected with the virus. This not only indicates a concern for personal health but also highlights the psychological impact of uncertainty and testing measures that can be perceived as invasive or stressful. Similarly, item 19, “I will go to practice/competition with fear”, with a loading of 0.750, shows how health-related fears extend into occupational and recreational activities. The fear of attending public events or group activities underscores concerns about exposure to the virus in closed or crowded spaces, which can significantly affect well-being and participation in usual activities. At the same time, item 16 (“I feel alone”), with a loading of 0.640, highlights the effects of social isolation. The pandemic has imposed restrictions on social interactions, exacerbating feelings of loneliness and separation from the social support network that is vital for mental health (Table 1).

Taken together, these articles reflect a wide range of negative emotions, from fear to loneliness, that are amplified by the pandemic context. Recognizing and addressing these feelings through psychological support programs and information campaigns could mitigate their impact on the mental health of the population. It emphasizes the need for an empathetic and proactive approach to public health crisis management, with a particular focus on emotional and psychological support for affected communities.

Factor 3 of the analysis, focused on substance use, highlights items that address the ways in which individuals managed the stress of the pandemic through potentially harmful behaviors. This factor shows that, in the face of a stressful global event, some individuals may resort to momentary solutions that may have long-term consequences for their health. Item 13, which reflects an increase in recreational alcohol consumption, with a loading of 0.782, and item 14, which indicates the use of mood-enhancing drugs, with a loading of 0.774, both emphasize the tendency of some individuals to use substances to cope with anxiety and uncertainty. These negative coping behaviors not only reflect the immense pressure felt by individuals but also the acute need for healthier support strategies. Using substances as a coping mechanism can provide temporary relief, but often at the cost of long-term health problems and addiction. Item 22, with a significant negative loading of −0.670, expresses respondents’ skepticism regarding the return to normality in the sports world. This pessimism may reflect a broad awareness of the lasting impact the pandemic is having on social and economic sectors, including sport, which is often vital to many people’s emotional and physical well-being (Table 1).

Interpreting these data in an integrated manner shows us that the impact of the pandemic on substance use behaviors is profound and complex. There is a clear need for targeted interventions that provide healthy coping alternatives, education about the risks of substance use, and support for those who have developed addictions in this stressful context. Also, addressing skepticism and uncertainty about the future of sporting activities and other key areas can help restore hope and reduce reliance on harmful stress management behaviors.

### 3.3. Correlation Analysis 

A detailed analysis of the correlations between depression and coping strategies used during the COVID-19 pandemic, as well as their impact on well-being, provides insight into the mechanisms by which individuals adapt to the stress generated by this global crisis. Table 2 shows the Spearman correlations that measure the links between depression levels and various coping strategies. For example, significant but low correlations are observed between depression and strategies such as rumination (0.244), perspective-taking (0.231), catastrophizing (0.266), and blaming others (0.247) (Table 2). These correlations suggest that coping strategies such as catastrophizing and rumination, which tend to accentuate the negative aspects of the current situation, are positively associated with higher levels of depression. In contrast, strategies such as positive reappraisal and planning focus show negative correlations with depression (−0.132 and 0.010, respectively), although these are not statistically significant. This could indicate that such positive strategies may help to manage stress more effectively but are not sufficient to significantly influence depression in the context of the pandemic.

In order to describe the relationships between depression and CERQ test variables, we analyzed the effectiveness of the regression model applied, so we obtained a determination coefficient of 0.131, which means that 13.1% of depression is determined by the developed coping strategies. The model of the coping mechanisms identified through CERQ predicts a variation in depression related to the COVID-19 pandemic in a percentage of 13% AdJR = 0.131. 

From the ANOVA calculation, we obtain F = 2.708 for a significant *p* = 0.008, which leads to the idea that the results are not due to a sampling error, and so the regression equation can be built in order to identify the coping mechanisms that are significant for depression. Table 3 details the results of an ANOVA analysis exploring the contribution of each coping strategy to depression levels. The analysis reveals non-significant coefficients for most coping strategies, except for perspective-taking and positive refocusing. These two strategies are the only ones to have significant regression coefficients (*p* = 0.019 for perspective-taking and *p* = 0.023 for positive refocusing), indicating a significant relationship with depression. Putting things into perspective (β = 0.278) appears to provide a benefit in managing depressed feelings, suggesting that reinterpreting negative events in a more positive or balanced way may help reduce depression. Positive refocusing (β = −0.321), which involves directing attention to positive or pleasant aspects, is also associated with a reduction in depression, reflecting the importance of shifting focus from negative to positive stimuli in managing well-being.

This finding highlights that coping strategies that encourage a more optimistic and balanced view of life situations may be particularly valuable in crisis contexts. Consequently, it is essential that intervention and psychological support programs integrate and promote these techniques, thereby providing affected individuals with the necessary tools to navigate the emotional challenges of the pandemic. This could include stress management workshops, solution-focused therapy, and counseling sessions to help individuals recognize and modify catastrophic or pessimistic thoughts. By building psychological resilience and promoting a more proactive and positive approach to adversity, these strategies can not only help reduce the incidence of depression during the pandemic but also improve long-term mental health, preparing the individual to cope better with other potential future crises.

### 3.4. Gender Variations and Coping Mechanism 

The detailed analysis of gender variations in depression and associated coping mechanisms presented in the paper explores how different coping strategies influence depressive states in men and women. These differences are highlighted by moderated regression analysis using the SPLIT FILE function, based on the method described by Aiken and West (1991). Regarding the SPLIT FILE analysis, the coping mechanisms identified by CERQ predict a variation in COVID-related depression in a proportion of 15% AdJR^2^ = 0.153 (f^2^ = 0.181) for the male sample and 10% AdJR^2^ = 0.100 (f^20^ = 0.111) for the female sample. This suggests that the identified coping mechanisms predict, to a greater extent, variations in depression in men compared to women. The associated F and p values indicate a lack of statistical significance (*p* > 0.05), suggesting that although there is a trend, it is not strong enough to be considered statistically significant (Table 4).

As can be seen in the ANOVA analysis (Table 5), the pattern made up of the coping mechanisms chosen by sex after the introduction of the moderator shows the lack of predictive significance of both models (*p* > 0.05 in both ANOVA analyses). However, by analyzing in more detail, certain patterns can be observed that the obtained results can indicate.

In the case of women, the positive refocusing mechanism shows significant predictive power, with a coefficient B of −0.488 and a t of −2.355, reaching significance at the *p* = 0.023 level. This suggests that women’s ability to redirect attention to the positive aspects of their lives has a strong and direct effect on alleviating depressive symptoms. This result could be influenced by women’s cultural tendency to value and engage in social relationships and emotional support, which are important external resources for protection against the negative effects of stressors. On the other hand, refocusing on planning is nearly significant, with a coefficient B of 0.341, a t of 1.996, and a *p* of 0.056. This indicates that women’s involvement in activity planning and proactive management of their time and tasks may contribute to a marginal reduction in depression, possibly due to the increased sense of control and personal efficacy this strategy provides.

For men, the results indicate no statistical significance for the coping mechanisms analyzed, suggesting that conventional coping strategies may not be as effective for them in managing depression in the context of the pandemic. This may be attributed to differences in emotional expression and use of support resources, which are often less pronounced in traditional male behavior.

It can be interpreted that women, compared to men, manage to reduce depression by refocusing on the positive aspects of their lives and seeking emotional and instrumental support from others, which we know is an important external resource to protect against the negative effects of stressors. Generally, this kind of behavior is part of a woman’s social role. Specifically, in Romania, women assume, to a greater extent, more traditional domestic roles than men, which gives them the opportunity to engage in useful, constructive activities with concrete results, refocusing on planning and keeping them away from rumination or other maladaptive strategies. Thinking about pleasant things rather than the actual negative experience is referred to as positive refocusing as a coping technique [32]. Mental disengagement, in the short term, might be a helpful reaction, but in the long run, it might prevent adaptive coping.

### 3.5. Sport Type Variations in Depression and Cognitive Schemas 

Analysis of variations in depression by type of sport played and associated coping mechanisms provides valuable insights into how the individual versus team sport context influences stress and depression management. Details in Table 6 and Table 7 are used to explore these differences, highlighting the significant impact of sport type on the effectiveness of coping mechanisms.

Table 6 presents the regression model summary for individual and team sports, illustrating how coping varies by sport context. The model for individual sports shows an adjusted R-squared of 0.221, indicating that approximately 22% of the variance in depression can be explained by coping mechanisms in this context, with a significant level of prediction (*p* = 0.034). This suggests that individual athletes may benefit more from certain coping strategies that are effective in reducing depressive symptoms.

In contrast, the model for team sports shows a negative R-squared adjustment (−0.017), indicating that coping mechanisms do not significantly impact variance in depression in this context, with a much lower level of significance (*p* = 0.541). This result may suggest that the dynamics and interactions specific to team sports may complicate the effectiveness of individual coping strategies, requiring different approaches to managing stress and depression.

It can be noted that the model of the coping mechanisms chosen by the individual athletes, after the introduction of the moderator, shows the significance of predicting the regression model for them only (ANOVA *p* < 0.05).

For the individual athletes, the positive reappraisal shows significant predictive power (B = −0.420, *t* = 2.308, *p* = 0.027). This predictor, giving the crisis some sort of positive significance, moderates depression in a good way. The coping mechanism of perspective has significant predictive power for depression (B = 0.392, *t* = 2.80, *p* = 0.036). For team athletes, no coping mechanism reached the level of significance necessary to be considered a strong predictor of depression. This could reflect the complexity of stressors in team sports, where success depends not only on individual performance but also on relationships and coordination within the team as well as external pressures.

Individual athletes showed fewer psychological changes during the epidemic, but team players faced more disruption to their training patterns and relationships with their teammates and trainers. The team players were particularly worried that their success relies on the team’s efforts and connections with others and, therefore, have developed more prominent signs of anxiety and depression [39,40]. The perceived loneliness and guilt feelings may have a negative impact on their quality of life, and consequently, the prevalence of sadness and depression in team sports may be higher [41], and their coping mechanisms to fight depression may be poorer. Individual athletes’ ability to experience positive emotions through the reappraisal of stressful situations can fight negative behavior in their case. However, according to Salles et al. [37], the personality traits and the genetic predispositions of the athletes are also important. 

## 4. Discussion

The findings of this study underline the significant impact of the COVID-19 pandemic on the mental health of sports students, highlighting crucial differences in coping mechanisms across genders and sports types. Depression, identified as a predominant psychological consequence in our study, showed a marked influence by gender and the type of sport, aligning with recent research that emphasizes the moderating role of these factors in psychological resilience during crises [58].

As previously presented by various authors [59,60], in the phase study, a significant rise in mental health issues was indicated due to the COVID-19 pandemic. Globally, anxiety and depression increased by 25% during the first year of the pandemic, underscoring the pervasive impact of the crisis on mental health [61]. This increase is attributed to the stress of social isolation, fear of infection, economic uncertainty, and disruptions in daily life during lockdowns. Importantly, the pandemic has highlighted substantial gaps in mental health services, which were exacerbated during this period of heightened need [62].

The use of healthy coping mechanisms and high levels of self-control were generally associated with lower levels of depression [63]. The depressive symptoms related to COVID-19 were higher among students because youth are already in complicated periods of life with intense transformations and commitments. The pandemic challenges have been added, which have exacerbated the existing stress [64]. More effective coping strategies, such as positive reappraisal or positive reframing, based on finding in every negative event some sort of positive significance, helped reduce stress [65]. 

The stress generated in the threatening context also had some positive effects on athletes’ lives. The literature review highlights that the interruption of training sessions and competitions led to a reduction in competitive tensions and expectations among the athletes, a decrease in the intensity of training, and the regeneration of their psychological resources [66,67]. Also, the athletes reported declines in rivalries and hostility, increasing the manifestation of resilience. The recovery time for injured athletes was extended, the time and quality of communication improved, and the exposure to the media and public was reduced. Coaches and the technical staff, if they manifested themselves as supportive and empathetic, constituted an important resource for athletes [68,69,70]. The media, which emphasized problems and mental imbalances, determined a reduction in stigma and labeling, normalizing psychological disorders and opening the way for therapeutic programs [71].

A negative impact was caused by exposure to social media with a constant stream of damaging COVID news or among people who were forced to isolate because they were infected [72,73,74,75]. Athletes’ commitment to performance declined dramatically, while the coach was perceived as a stressor because, in many cases, he continued to put pressure on home-training athletes in difficult conditions and had expectations about the results. Also, the courses and exams held in the virtual environment brought additional sources of stress to the student-athletes participating in the study [76,77].

Men and women exhibited distinct coping mechanisms, which may explain the variability in depression scores. Women utilized more emotion-focused coping strategies, such as seeking social support, which is consistent with previous findings [32]. Conversely, men often relied on avoidance strategies, which might not be as effective in the long-term management of depressive symptoms [63]. The differential engagement with coping strategies by gender underscores the need for gender-sensitive approaches in psychological interventions and support. On the other hand, men were more affected by substance use, as shown in the studies of Baker et al. [78], Luchetti et al. [79], and the World Health Organization [80].

The type of sport played a pivotal role in how athletes coped with the pandemic-induced stress. Athletes in individual sports are generally more on their own, and the social distance during lockdown was not a major problem, as it was for the team players, as shown by Ntoumanis et al. [52]. Studies conducted in August 2020 on athletes worldwide showed that the highest number of infections were recorded in football and basketball, which raised concerns about the possibility of illness among team players [41,81,82]. After the competition reopened, team athletes felt less prepared, with much less homogeneity and cohesion on the team level and a loss of motivation, meaning, and identity by participating in competition without public and emotional support [83,84].

While the negative impact is evident, there are also reports suggesting that some athletes have successfully used this period for recovery and mental health improvements, facilitated by support from mental performance consultants and access to psychosocial services [85]. This highlights the heterogeneity of experiences and the potential for resilience-building strategies that can mitigate the adverse effects of such global disruptions. Effective coping mechanisms during the pandemic have included structured training programs, virtual engagement with teams, and increased mental health resources. These interventions have been crucial in maintaining athletes’ mental well-being and should be incorporated into ongoing support systems [86].

Even though we are in a post-COVID era, it is essential to focus on the coping strategies that have been effective for performance athletes during the lockdown period. They can add a new layer to the psychological preparation of athletes and serve as a guide for future crises, benefiting both performance athletes and students.

Coping strategies that worked during the lockdown, such as training adapted to home conditions, online psychological support, and mindfulness techniques, should be integrated into the athletes’ regular routines to improve their psychological resilience. In addition, they can be used to develop more robust mental training programs to help athletes better manage stress during competitions and quickly adapt to unexpected changes in their sports schedule [87]. Lessons learned from the pandemic can also serve as a foundation for creating guidelines and protocols for managing future crises. These protocols could include anticipatory psychological preparation and adaptation to crisis scenarios, essential not only for athletes but also for students facing similar pressures in competitive academic environments [49,88].

Therefore, it is crucial that we continue to harness and refine these coping strategies to support not only athletic performance but also the mental health of students and performance athletes, preparing them for future challenges [89]. This holistic approach can help create a solid foundation for mental and physical well-being in the face of ongoing uncertainties.

## 5. Conclusions

This study’s results indicate significant psychological impacts of the COVID-19 pandemic on sports students, demonstrating the pivotal role of gender and type of sport in the modulation of depressive symptoms. Our findings underscore that depression was the most prevalent psychological issue among the participants, with noticeable differences in coping strategies influenced by gender and the sports discipline involved.

The data revealed that gender plays a crucial role in how depression manifests and is managed. Female participants reported greater utilization of coping strategies like positive refocusing and perspective-taking, which were linked to lower depression scores. These strategies, involving shifting focus towards more positive or balanced perspectives, appear more effective in mitigating the psychological toll among female athletes.

Differences based on the type of sport were also prominent, with individual sports athletes showing a better ability to manage stress through adaptive coping mechanisms compared to their team sports counterparts. The results suggest that the solitary nature of training in individual sports might contribute to a better-developed resilience to the isolating conditions of the pandemic.

A concerning finding was the correlation between increased feelings of hopelessness and a higher susceptibility to substance use. The results show more severe psychological distress could lead to unhealthy coping mechanisms, highlighting the need for targeted mental health interventions in this demographic. At the same time, the necessity of structured mental health support tailored to the unique needs of sports students is highlighted, particularly considering the distinct impacts of gender and sport type. Intervention programs should incorporate strategies proven effective in these subgroups, such as positive reappraisal and active planning, to enhance resilience and psychological well-being.

Further research is needed to explore the long-term effects of the pandemic on this population, with a focus on developing and testing interventions that address the specific challenges identified. Longitudinal studies could provide deeper insights into the persistence of these psychological effects and the efficacy of different coping strategies over time. Finally, it is essential to collaborate with sports psychologists, coaches, and the athletes themselves in designing these studies and interventions. Their input can ensure that the strategies are practical, culturally sensitive, and aligned with the athletes’ specific needs and the realities of their sports. This collaborative approach can significantly enhance the practical applicability and impact of the research findings, providing athletes with the tools they need to thrive in both their professional and personal lives, even in the face of future global disruptions.

This study acknowledges several limitations that may affect the interpretation and generalizability of the findings. The reliance on self-reported data introduces potential biases, such as social desirability and recall bias, which can impact the accuracy of the results. Additionally, the absence of pre-pandemic baseline data limits the ability to fully assess the impact of COVID-19 on athletes’ mental health and coping strategies, making it difficult to determine the extent of changes attributable to the pandemic. Moreover, the correlational nature of the study prevents causal inferences, as significant relationships between variables cannot confirm causality. Future research should incorporate objective measures, include longitudinal designs, and explore causal relationships to enhance the robustness and applicability of the findings, providing a clearer understanding of the psychological impact of global crises on athletes.

## Figures and Tables

**Table 1:** Survey item values based on factorial analyses.

Rotated Component Matrix ^a^	Component		
	1	2	3
4. Since the pandemic, I have been nervous	0.791	−0.011	0.043
3. I have been feeling depressed lately	0.758	0.054	0.189
1. I feel hopeless since the pandemic	0.663	0.080	0.125
5. Watching COVID news causes me agitation	0.573	0.298	0.003
2. I feel tired even though I stay at home more than before	0.539	0.054	−0.036
12. I had difficulty sleeping during this period	0.412	0.260	0.041
21. I am concerned about the idea of testing for COVID-19	−0.062	0.789	0.114
19. I will go with fear to training/competitions	0.063	0.750	0.105
16. I feel alone	0.337	0.640	0.063
20. Perceived risk of getting sick according to gender	−0.051	0.435	−0.001
13. I drank more alcohol to relax	0.049	−0.023	0.782
14. I used medication to get a good mood	0.118	0.217	0.774
22. Sport will return to normal after the pandemic	−0.040	−0.069	−0.670
15. I had difficult relationships with family during lockdown	0.232	0.101	0.466
11. I had food problems during the lockdown	0.198	0.132	0.225
17. I felt detached from colleagues and friends	0.335	0.402	−0.033
6. My physical condition deteriorated	0.213	−0.082	−0.235
8. Sport is vulnerable to crises and disasters	0.082	0.037	−0.001
7. My sports career was put in jeopardy because of the training breaks	0.346	0.180	0.094
10. The media and the public lost interest in sports during the crisis	0.192	0.130	−0.018
9. This year’s competitions are compromised	0.064	−0.109	0.130
18. The amount of time on social media according to gender	0.195	−0.005	0.069
17. I felt detached from colleagues and friends	0.335	0.402	−0.033

Extraction Method: Principal Component Analysis; Rotation Method: Varimax with Kaiser Normalization; ^a^ Rotation Converged in 10 Iterations.

**Table 2:** Spearman’s correlations between depression and coping strategies.

Spearman’s Correlations											
		Depression	Self-b ^2^	Accept ^3^	Rum ^4^	Pos.rea ^5^	Ref.plan ^6^	Pos.ref. ^7^	Put.per ^8^	Catastr ^9^	Blm.oth ^10^
Depression	Cc ^1^	1.000	0.129	0.083	0.244 *	−0.132	0.010	−0.135	0.231 *	0.266 **	0.247 *
	Sig. (2-tailed)		0.193	0.403	0.013	0.183	0.920	0.175	0.019	0.007	0.012
	N	103	103	103	103	103	103	103	103	103	103

* Correlation is significant at the 0.05 level (2-tailed); ** correlation is significant at the 0.01 level (2-tailed). ^1^ correlation coefficient; ^2^ self-blaming; ^3^ acceptance; ^4^ rumination; ^5^ positive reappraisal; ^6^ refocusing on planning; ^7^ positive refocusing; ^8^ putting into perspective; ^9^ catastrophizing; ^10^ blaming others.

**Table 3:** ANOVA analysis of depression variations related to coping strategies.

ANOVA Analysis of Depressions’ Coefficients ^a^									
		Unstandardized Coefficients		Standard-Ized Coef-Ficients	t	Sig.	Correlations		
		B	Std. Error	Beta			Zero-Order	Partial	Part
1	(Constant)	10.837	1.716		6.316	0.000			
	Self-blame	0.050	0.266	0.022	0.190	0.850	0.143	0.020	0.018
	Acceptance	0.081	0.292	0.032	0.276	0.783	0.097	0.029	0.025
	Rumination	0.306	0.283	0.122	1.080	0.283	0.224	0.111	0.100
	Positive reappraisal	−0.411	0.247	−0.173	−1.664	0.099	−0.120	−0.170	−0.154
	Refocus on planning	0.262	0.326	0.102	0.804	0.424	0.020	0.083	0.074
	Positive refocusing	−0.764	0.330	−0.321	−2.315	0.023	−0.124	−0.233	−0.214
	Putting into perspective	0.675	0.284	0.278	2.378	0.019	0.196	0.239	0.220
	Catastrophizing	0.352	0.293	0.135	1.202	0.232	0.259	0.124	0.111
	Blaming others	0.228	0.245	0.098	0.929	0.355	0.224	0.096	0.086

^a^ Dependent Variable: Depression; β—Test; Std. E—Standard Error; t—Student’s *t*-test; Sig.—Significance.

**Table 4:** The SPLIT FILE analyses depression variations according to gender and related to coping mechanisms.

Model Summary										
Gender	Model	R	R Sq	Adj R Sq	Std. E	Change Statistics				
						R Sq Ch	F Ch	df1	df2	Sig. F Ch
Male	1	0.553	0.306	0.153	4.172	0.306	2.007	9	41	0.063
Female	1	0.509	0.259	0.100	4.505	0.259	1.630	9	42	0.138

R—R Test; RSq—R Squared; Adj R Sq—Adjusted R^2^; Std. E—Standard Error; RSqCh—R Square Change; FCh—F Test Change; df1, 2—Differences; Sig. FCh—Significance of F Test Change.

**Table 5:** ANOVA analysis of depression variations according to gender and related to coping mechanisms.

ANOVA ^a^							
Gender	Model		Sum Sq	df	Mean Sq	F	Sig.
Male	1	Regression	314.359	9	34.929	2.007	0.063 ^b^
		Residual	713.563	41	17.404		
		Total	1027.922	50			
Female	1	Regression	297.736	9	33.082	1.630	0.138 ^b^
		Residual	852.321	42	20.293		
		Total	1150.058	51			

^a^ Dependent Variable: Depression; ^b^ Predictors: (Constant), Blaming others, Positive reappraisal, Self-blaming, Catastrophizing, Rumination, Acceptance, Putting into Perspective, Refocusing on Planning, Positive Refocusing; Sum Sq—Sum of Squares; Mean Sq—Mean of Squares; F—F Test; Sig—Significance.

**Table 6:** The SPLIT FILE analyses depression variations according to type of sport and related to coping mechanisms.

Model Summary ^a^										
Type of Sport	Model	R	R Sq	Adj R Sq	Est. Std. E	Change Statistics				
						R Sq Ch	F Ch	df1	df2	Sig. F Ch
Individual	1	0.620 ^b^	0.384	0.221	4.325	0.384	2.358	9	34	0.034
team	1	0.375 ^b^	0.140	−0.017	4.551	0.140	0.890	9	49	0.541

^a^ Dependent Variable: Depression; ^b^ Predictors: (Constant), Blaming Others, Acceptance, Positive Reappraisal, Catastrophizing, Rumination, Putting into Perspective, Self-blaming, Refocusing on Planning, Positive Refocusing; R—R test; RSq—R Squared; Adj R Sq—Adjusted R^2^; Std. E—Standard Error; RSqCh—R Square Change; FCh—F Test Change; df1, 2—Differences; Sig. FCh—Dignificance of F Test Change.

**Table 7:** ANOVA analysis of depression variations according to type of sport and related to coping mechanism.

ANOVA ^a^							
Type of Sport	Model		Sum of Squares	df	Mean Square	F	Sig.
Individual	1	Regression	396.944	9	44.105	2.358	0.034 ^b^
		Residual	635.965	34	18.705		
		Total	1032.909	43			
Team	1	Regression	165.807	9	18.423	0.890	0.541 ^b^
		Residual	1014.736	49	20.709		
		Total	1180.542	58			

^a^ Dependent Variable: Depression; ^b^ Predictors: (Constant), Blaming Others, Putting into Perspective, Self-blaming, Rumination, Refocusing on Planning, Positive Reappraisal, Acceptance, Catastrophizing, Positive Refocusing; Sum Sq—Sum of Squares; Mean Sq—Mean of Squares; F—F test; Sig—Significance.

## Data Availability

The raw data supporting the conclusions of this article will be made available by the authors upon request.
